# Books and Authors

**Published:** 1910-09

**Authors:** 


### BOOKS AND AUTHORS

**American Practice of Surgery.** A complete System of the Science and Art of Surgery by representative surgeons of the United States and Canada. Edited by **Joseph D. Bryant,** M.D., and **Albert H. Buck,** M.D., New York. Complete in eight volumes. Vol. VII. Regional Surgery. Imperial octavo, pp. 961. Profusely illustrated. New York. William Wood & Company. 1909. (Price, cloth, $7.00.)

A continuation of regional surgery furnishes the theme of this volume, a topic that is of the deepest interest to the practical surgeon. Indeed, the two volumes, the sixth and seventh, dealing with this subject are not second in importance to any volumes in the series, nor to any similar works that have been issued relating to regional surgery anywhere or in any tongue. The contributors in this, as in the former volumes, have been well chosen, being men of distinction for the most part in their several fields. The names found among them include E. Wyllis Andrews, J. D. Griffith, Nathan Jacobson, William M. Masten, Andrew J. McCosh (deceased), Albert J. Ochsner, and James P. Tuttle. The other contributors are men of high standing though perhaps with less extended fame; but the material they present is not less scientific nor less valuable.

Many of the subtopics presented in this book will not be found elsewhere, which does not mean that they are new absolutely, but indicates that they have not found their way into textbooks or even into smaller works. Masten's article on abdominal section is quite new, and the topic is dealt with in an interesting as well as scholarly fashion. He refers to several terms that have been proposed as substitutes for abdominal section which was first suggested and employed by Lawson Tait. We have condemned "celiotomy" as a useless if not improper term, and we note that Masten does not approve of any of the substitutes yet proposed for abdominal section. He quotes Dr. Frank P. Foster, an undisputed authority, in criticism of "celiotomy." Strictly speaking, abdominal section would apply only to the cutting through the parietes, but as at present understood it means the whole operation, including whatever may be done to the viscera. The operation may be exploratory or actual, and the technic is here given applicable to either; also the post-operative care is described. Altogether this monograph is full of interest and merits the careful attention of every abdominal surgeon.

The surgery of the pericardium, heart, and bloodvessels, by Le Conte and Stewart is one of the longest sections of the work, containing about 185 pages. The increasing interest developing in this class of surgery, gives zest to this section, one which these authors have handled masterfully.

Jacobson's article on tuberculous peritonitis is a scholarly presentation of this important topic. The references are extensive, covering the literature up to the immediate present, and making the article one of supreme value.

The article on surgical diseases of the appendix, written by Andrew J. McCosh, was completed only a few days before his tragic death. It is replete with interest. The references were supplied by the editors. The article on hernia (Andrews), the one on surgical diseases and wounds of the intestines (Oviatt), and that on surgical diseases of the anus and rectum (Tuttle and Earle), are each comprehensive presentations of the respective topics mentioned. They are all admirably illustrated and are valuable additions to the literature of the subjects with which they deal. We inadvertently omitted to mention in this group Ochsner's disquisition on surgical diseases of the stomach and esophagus, which is an able monograph, and gives the best and latest information on a surgical question of growing interest and importance.

The volume is everything to be desired not only in the quality of its literature but in illustrations and general make-up. It will stand as authority in the topics it presents, for many years to come.

---

**International Clinics.** A Quarterly of Illustrated Clinical Lectures and especially prepared articles on Treatment, Medicine, Surgery, Neurology, Pediatrics, Obstetrics, Gynecology, Orthopedics, Pathology, Dermatology, Ophthalmology, Otology, Rhinology, Laryngology, Hygiene, etc. Edited by **Henry W. Cattell,** M.D. Vol. II, twentieth series. Philadelphia and London: J. B. Lippincott Co. 1910. (Cloth, $2.00.)

Under the head of diagnosis and treatment in this number we note five articles, two of which are of special importance—namely, one on the treatment of cardiovascular disease, by James Tyson; and one on the diagnosis of chronic pancreatitis, by Charles Greene Cumston. Tyson discourses favorably upon the bath treatment, Neuheim and other German baths, in certain approved cases of cardiovascular disease where such non-medicinal measures are available; and among diuretics, when medicinal treatment becomes necessary, he places infusion of digitalis leaves (from good leaves) in the first rank. We have long been attached to this oldfashioned remedy for the purpose named.

In the group under medicine we observe three articles, one each by Frank Billings, James J. Walsh, and S. R. Klein. Under a special head, "Cancer," we find an interesting as well as an instructive article by J. Garland Sherrill (Louisville), in which he discourses upon so-called inoperable tumors, especially of the parotid gland. He relates several illustrative cases upon which he operated successfully and photographs showing the result in one instance are introduced.

Surgery offers but two articles, the first by John B. Deaver and the second by Louis Fischer. Deaver's number is a resume of twenty-six operations at Saturday clinics given by him at the German Hospital, Philadelphia, of which he is surgeon-in-chief. The number of patients operated was 234 among which were 100 cases of appendicitis, acute and chronic; 13 cases of cholelithiasis; two of duodenal ulcer; and two of chronic pancreatis. The entire report is of interest, being presented in an attractive manner by a master.

John W. Ballantyne, Edinburgh, in the department of obstetrics, presents an excellent article on the contingent treatment of eclampsia. It is one of the clearest statements we have seen regarding the management of this direful complication of pregnancy, labor, or the puerperium. Robert Jardine (Glasgow), offers also a paper on the treatment of eclampsia, and William B. Doherty publishes one with the strange title "cyesiognosis."

One article is published under each topic of dermatology, pediatrics, and neurology; one article on book-plates, and one on the socalled medical homecoming week at the University of Pennsylvania. This event was celebrated during the week beginning with Easter Monday, 1910, by a series of post-graduate lectures. These are edited by John G. Clark. This volume will take rank as one of the more interesting numbers of the series.

---

**Diseases of Infancy and Childhood.** By **Henry Koplik,** M.D., Attending Physician to the Mount Sinai Hospital, New York. Third edition. Octavo, pp. 944. Illustrated. New York and Philadelphia: Lea & Febiger. (Cloth, $5.00.)

Four years ago (September, 1906) we published a notice of the second edition of this superb work in which we said that Koplik was not a new name nor was he a new author to become acquainted with. His first edition was received with encomiums and his fame as an accomplished pediatrist is world-wide. His treatise takes rank with the best in any land or tongue and his ability as a teacher is acknowledged everywhere.

In this edition the advances that have taken place in these intervening years are recorded, a careful revision having been made. Much has been eliminated, more has been added, and many chapters have been recast or rewritten. The advances in pathology, diagnosis, and treatment in the last few years have clarified many hitherto obscure points that will be found well presented in this new issue. Among the many changes of high interest and importance may be mentioned the improvements in the diagnosis and treatment of the infections, the latest advances in infant feeding, the management of gastric and nervous diseases, including the new treatment of meningitis, the chapters on cystitis and pyelitis, and the new sections on tuberculosis, idiocy, dwarfism and neurotic conditions. A feature has been made of improved methods of examination and therapy. As young children cannot aid the diagnostician by describing their symptoms, much depends on their expression.

We must mention the illustrations as being of high-class. Some of the colored plates are superb, notably the one depicting tonsilar diphtheria. It is a treatise of value to both pediatrist and general practitioner.

---

**The Optic Nerve and the Accessory Sinuses of the Nose.** By **Professor A. Onodi,** Budapest, Member of the Hungarian Academy of Sciences. Translated by **J. Luckoff,** M.D., Cape Town. Octavo, pp. 101. With 50 illustrations. New York: William Wood & Co. 1910. (Price, $3.50 net.)

Ophthalmologists everywhere will be glad to possess this work. In it the minute anatomy of the nasal accessory sinuses and their relations to the optic nerve are thoroughly worked out, —a study that has engaged the author's attention for more than ten years. Laryngologists and rhinologists, too, will be pleased to have access to such a beautiful anatomic portrayal of the immediate environment of the principal fields of their work. The fifty illustrations are superb, forty-nine of which have never been excelled,—we might have said, with truth, never equaled —for artistic beauty and anatomic accuracy. The descriptive text is clear, giving a knowledge of the topographic anatomy that cannot fail to be understood and remembered. The thirty pages of clinical considerations, too, are full of interest to specialists in particular. The entire work is a contribution to scientific accuracy that will stand the test of time.

---

**The Examination of the Function of the Intestines by means of the Test Diet.** By **Professor Adolf Schmidt,** Physician-in-Chief of the City Hospital, Friedrichstadt in Dresden. Translated by **Charles D. Aaron,** M.D., Professor of Diseases of the Stomach and Intestines in the Detroit Post-graduate School of Medicine. Second edition. 12 mo., pp. 133. Philadelphia: F. A. Davis Co. (Cloth, $1.00.)

The favorable impression created by this brochure in its first edition led to its early exhaustion. The author is well known in both hemispheres as an expert in this topic, in which, also, he is a pioneer. The translator, too, is a gastroenterologist of established reputation, formerly a pupil of Professor Schmidt and a graduate of the University of Buffalo. He is now Professor of Diseases of the Stomach and Intestines at the Detroit Post-Graduate School of Medicine, and Clinical Professor of Gastroenterology at the Detroit School of Medicine. New material has been incorporated in this edition, particularly regarding those disturbances of the intestines due to perversion of the function of the stomach, liver, and pancreas. It may be regarded as representing the most advanced thought on the application of the test diet to diagnosis and treatment.

---

**Pocket Therapeutics and Dose-Book.** By **Morse Stewart,** Jr., B.A., M.D. Fourth edition. Small 32 mo. of 263 pages. Philadelphia and London: W. B. Saunders Company, 1910. (Cloth, $1.00 net.)

It is important to write prescriptions correctly as well as plainly, in order that no mistake may be made in compounding them. This little book gives instructions on this topic which should be followed with accuracy. The dose table is a useful reminder, especially regarding those remedies of infrequent employment, hence likely to be forgotten as to dosage. This edition, the fourth, finds the book carefully revised, obsolete remedies having been eliminated and newer ones entered in their place. It now represents practically every drug in use,—those already approved and those on trial.

---

**Essentials of Laboratory Diagnosis.** By **Francis Ashley Faught**, M.D., Director of the Laboratory of Clinical Medicine, Medico-Chirurgical College, Philadelphia. 12 mo., pp. 317. Illustrated. Philadelphia. F. A. Davis Co. (Cloth, $2.00.)

In these days when laboratory methods play such an important part in diagnosis, it becomes a necessity for every student and junior physician to possess a book of this nature,—a book giving the essentials, and only the essentials, of procedures requisite to enable one to apply laboratory methods to clinical diagnosis. The author has chosen well his material, and has made a compact book that gives simple and reliable processes through which the required information may be obtained, without sacrificing valuable time to uncertain or less understood ways. Concise anatomical methods are presented in the latest light, and at the end an appendix gives a plan for the equipment of a clinical laboratory, which cannot fail to become useful to many students and medical men. Nothing more useful on this topic has appeared from the press up to the present time.

---

**A Textbook of Pathology.** By **Joseph McFarland**, M.D., Professor of Pathology and Bacteriology in the Medico-Chirurgical College of Philadelphia. Second edition. Octavo of 856 pages, with 437 illustrations, some in colors. Philadelphia and London. W. B. Saunders Company, 1910. (Cloth, $5.00; half morocco, $6.50, net prices.)

Pathology doesn't change in its manifestations, but our knowledge of it does, hence the necessity of revising textbooks that deal with it at short intervals. McFarland has long been recognised as a teacher of a high order, as well as an authority in the front rank on this topic. He perhaps is best known through his work on pathogenic bacteria, which already has passed through its sixth edition. Revision of the present treatise has been delayed beyond the desire of the author; nevertheless, he has revised it now, page by page, leaving nothing to be desired from carelessness or neglect. This has enabled him to incorporate any advances of value and to eliminate errors as well as to supply omissions, which makes it now one of the completer and better textbooks extant on pathology. It is copiously illustrated with useful cuts in black and colors.

**Physiology and Pathology of the Semicircular Canals.** By **Adolph E. Ibershoff,** M.D. 12 mo, with 8 illustrations. New York: Paul B. Hoeber. 1910. (Cloth, $1.00.)

This essay gives a condensed statement or excerpt of the clinical studies of Barany on the semicircular canals. The value of nystagmus as a diagnostic aid in labyrinthine disease is pointed out and dwelt upon at considerable length. The entire brochure is instructive and of special value to the aurist. Dr. Ibershoff is a man of scientific attainments and an enthusiast in his work. He has performed a distinctive service by rendering the clinical studies of Barany available to the English speaking profession.

---

**Johns Hopkins Hospital Reports.** Vol. 15. Baltimore. The Johns Hopkins Press. 1910.

This volume contains material relating to studies in internal medicine and to skin grafting,—fifteen articles in all. The topics on internal medicine pertain to diarrhea in children, lobar pneumonia, terminal pneumonia, endocarditis and pericarditis complicating pneumonia, empyemia in acute lobar pneumonia, venous thrombosis in pneumonia, pneumococcic arthritis, pneumococcic meningitis, delayed resolution in lobar pneumonia, and serum therapy in epidemic cerebrospinal meningitis. The single article on skin grafting gives an elaborate review of 550 cases of skin transplantation which is well illustrated.

---

**Prescription Writing and Formulary.** By **John M. Swan,** M.D., Associate Professor of Clinical Medicine, Medico-Chirurgical College of Philadelphia. 32 mo, of 185 pages. Philadelphia and London: W. B. Saunders Company, 1910. (Flexible leather, $1.25 net.)

Prescription writing is an accomplishment that should be acquired by every physician; it is an art, too, the lack of which may betray ignorance to a multitude of people. A reputation may be made or marred by the construction of a prescription. This book carefully studied and heeded will enable one to avoid the faults and errors of careless prescription writing. That part of the book allotted to the formulary contains 1043 prescriptions, that may become of use to those who desire stereotype reminders of clinical therapeutics; perhaps, also, to those beginning to learn how to write prescriptions they may serve as useful models; but ready made prescriptions, as a rule, are to be deprecated. This formulary, however, contains some that are practical and therefore of advantage.

---

**Handbook of Electro-Therapeutics.** By **William James Dugan,** M.D., Lecturer on Electro-Therapeutics in Jefferson Medical College, Philadelphia. Illustrated. Octavo, pp. 242. Philadelphia: F. A. Davis Company. 1910. (Cloth, $2.00.)

This book, which the author says is intended for students and physicians who know comparatively little about electricity or electrical apparatus, appears to fulfil admirably the purposes for which it is planned. It is concise in its method of dealing with the subject, yet sufficiently comprehensive for practical purposes. It instructs with reference to the apparatus to be selected, and then tells how to use it. A chapter on magnetism, which is quite new, explains the relationship between electricity and magnetism. No doubt there is a place for such a handbook, and Dugan seems to have supplied a necessity that heretofore has existed in this regard.

